# Impacts of Emergency Treatments on Sediment Microbial Communities Following Sudden Thallium Contamination Events: A Microcosm Study

**DOI:** 10.3390/microorganisms13061336

**Published:** 2025-06-09

**Authors:** Xiaodie Cai, Zeqiang Huang, Sili Chen, Zhengke Zhang, Jingsong Wang, Xinyu Wen, Yuyin Yang

**Affiliations:** 1 School of Civil Engineering, University of South China, Hengyang 421001, Chinaaragorn0917@foxmail.com (Z.H.);; 2 South China Institute of Environmental Sciences (SCIES), Ministry of Ecology and Environment (MEE), Guangzhou 510655, China

**Keywords:** microbial communities, biogeochemical processes, emergency remediation, Thallium

## Abstract

Following heavy metal pollution caused by thallium in watersheds, people typically employ emergency treatment methods such as water sampling and transfer for dilution or in situ coagulation and precipitation. However, the thallium that is adsorbed by the precipitates in the sediment persists for a long time and is gradually released, posing a significant threat to the ecosystem. In this study, the 16S rRNA sequencing method was used to simulate the effects of water dilution or in situ coagulation and precipitation on microbial communities through thallium impact loading and thallium-containing iron floc shaking bottle experiments. The emendation of Fe(III) floc led to an increase in the relative abundance of Actinobacteriota. Meanwhile, *Nitrospira* and Proteobacteria exhibited distinct tolerances to Tl shock and Tl floc stress, respectively. Thallium pollution inhibited the reduction in nitric oxide and nitrogen fixation while increasing the relative abundance of the napA/B genes and decreasing the relative abundance of narG/H genes involved in nitrate reduction. This study offers new insights into the effects of various emergency treatment measures on river ecosystems following sudden thallium pollution, particularly from the perspective of microbial community composition and biogeochemical cycles.

## 1. Introduction

Thallium (Tl) is a trace element in the environment that presents strong toxicity to animals, plants, humans, and microorganisms [1,2]. Tl has dual geochemical properties of both lithophilicity and sulfur affinity and is often associated with cloud sulfide minerals, potassium minerals, etc. [3,4]. During the utilization of associated minerals, Tl was introduced to wastewater unconsciously and then entered into the water environment due to the intentional illegal discharge of wastewater or water treatment failure for Tl [5,6].

In recent years, there have been multiple incidents of Tl pollution in rivers around the world. Tl pollution incidents have shown a recurring pattern of high incidence in certain industrially intensive regions, particularly in non-ferrous metal mining areas [7,8]. Notable examples include the Tl pollution incident in the Canon River in the UK in 2012, a Tl exceedance incident in Hejiang, Guangxi, China, in 2013, a pollution event in the Bacatoyo River Basin in Italy in 2017, and another pollution incident in the Jialing River (Guangyuan section) in Sichuan Province, China, also in 2017 [6,9,10]. Tl can accumulate in algae and sediments and can be concentrated and transported through the food chain, seriously damaging the health of aquatic ecosystems. This Tl spill posed a serious threat to the water safety of the surrounding residents as well as the health of river ecosystems [11,12,13].

In the event of a sudden Tl water pollution incident, common emergency response measures include water diversion dilution and chemical coagulation sedimentation [14,15]. Emergency response measures for water diversion and dilution effectively address pollution by introducing clean water to mix with the contaminated water, rapidly diluting the Tl concentration to a safe threshold [16]. Chemical coagulation sedimentation involves transforming Tl into solid particles (flocs) through a process called flocculation, which is then allowed to settle [12,17]. This method can significantly lower the pollution concentration in aquatic systems. However, the Tl-containing flocs that accumulate in sediments may release Tl back into the water due to disturbances like water flow or activity from bottom-dwelling organisms, which poses potential ecological risks [12,18]. Therefore, it is crucial to further investigate the specific factors and mechanisms that influence the ecological effects of various emergency response measures when addressing a sudden Tl pollution incident in a watershed.

Microorganisms play a vital role in the cycling of materials and energy metabolism within river ecosystems [19]. Notably, unlike other organisms, such as animals and plants, microbial communities exhibit greater sensitivity to environmental disturbances, making them significant biological indicators for assessing the health of aquatic ecosystems [20]. Existing studies have initially investigated the impact of sudden Tl leakage in river basins on sediment microbial communities. The research findings indicate that Tl toxicity substantially diminishes bacterial diversity in sediments [21]. In addition to instances of abrupt Tl discharge, varying levels of Tl contamination have been found to substantially impact both the diversity and structure of microbial communities in various pollution scenarios, including those in agricultural lands and mining sites [22,23].

Microorganisms are key drivers of element cycling, including the cycling of carbon, nitrogen, and sulfur within ecosystems [24,25]. Therefore, exploring the effects of Tl leakage on the microbial regulation of these processes is crucial for understanding how river systems and broader ecosystems respond. In the Tl contamination incident in the Paosha River, the total abundance of nitrogen-cycling genes increased in the Tl-contaminated samples [21]. This suggests that Tl leakage may enhance nitrogen cycling. Research by Yan et al. in areas affected by sudden Tl pollution shows that Tl stress upregulates genes related to the initial nitrogen fixation pathway and the dismutated nitrate reduction pathway [26]. Tl pollution causes significant changes in microbial communities as they respond to heavy metal stress, disrupting the balance of nitrogen and sulfur cycles in the original river ecosystem. However, studies on the biogeochemical responses to Tl contamination are still relatively limited.

This study simulates contamination scenarios under controlled laboratory conditions to investigate the impacts of Tl shock loading and Tl-bearing flocs on microbial communities and biogeochemical cycling processes. By integrating these approaches, we systematically dissected the response variations in microbial assemblages under different emergency treatment strategies and identified the key driving factors governing community dynamics.

## 2. Materials and Methods

### 2.1. Microcosm Setup

Environmental samples for microcosm setup were collected from the Yexi River, Jiangxi Province, China. This region has long been threatened by the heavy metal smelting industry and experienced a sudden Tl pollution event in 2022 [27]. Environmental water and sediment samples were collected following the method described in the previous literature [28] and were transported to the laboratory in 24 h for subsequent experiments. The Tl concentration in the collected clean river water was measured at 0.02 μg/L, which is below the limit of 0.1 μg/L set by China’s Environmental Quality Standards for Surface Water (GB 3838-2002 [29]). In the sediment, the Tl concentration was recorded at 0.3 mg/kg, which is also below the threshold of concern recommended by the European Union’s Water Framework Directive (WFD). The other standard physicochemical indicators of the samples are recorded in Supplementary Table S1. Industry wastewater containing high concentrations of Tl was preserved from a previous pollution incident and was used for contamination simulation. The concentration of other metal(loid)s in the wastewater met the requirements of national standards.

In the experiment, 130 g of sediment was combined with 170 mL of clean river water in a 500 mL conical flask. The dynamic effects of various emergency treatment methods on the microbial community were simulated under controlled laboratory conditions that mimicked pollution. In addition to an uncontaminated blank control group (BLK), five simulation scenarios were established as follows:

The Tl Shock Load Group (POLL, POLH): High and low concentrations gradients of Tl were introduced over the water, with concentrations set at 50 μg/L (POLL) and 200 μg/L (POLH), respectively. Due to the implementation of a simulated water diversion dilution as an emergency measure, the pollutant concentrations were not sustained for extended periods. After a 2-day incubation period, the contaminated water was replaced with clean river water.

The Tl-Free Flocs Group (BLKF): A total of 600 mL of clean river water was treated by adjusting the pH to a range of 8.0 to 9.0, followed by the addition of 3 mL of a 10 g/L ferric sulfate polymer solution. The mixture was stirred at a rate of 200 revolutions per minute (r/min) for 3 min, then at 40 r/min for 15 min, and was allowed to settle for 30 min. The iron flocs, which were free of Tl, were then collected. This procedure was repeated ten times, and the collected flocs were added to an uncontaminated microcosm system.

The Coagulation Tl-Containing Flocs Group (EMEF): For this group, river water contaminated with Tl at a concentration of 200 μg/L was used instead of clean water. All other steps followed the same procedure as the BLKF group.

Pre-Oxidation—The Coagulation Tl-Containing Flocs Group (EMEOF): In this variation, the pH of the river water contaminated with Tl at a concentration of 200 μg/L was adjusted to a range of 8.0 to 9.0. After this adjustment, 1 mL of a 1 g/L potassium permanganate solution was added for pre-oxidation, which lasted for 30 min. Following this step, the process continued according to the procedures outlined for the BLKF group.

Detailed information regarding the coagulation treatment is listed in Text S1. All of the microcosms were incubated at 25 °C and 120 rpm, with three biological replicates for each treatment.

### 2.2. Sequencing and Data Processing

The amplicon sequencing was carried out on an Illumina MiSeq PE 250 platform at the V4–V5 region of the bacterial 16S rRNA gene and was amplified with primer sets 515F and 907R [30]. Multiplexed paired-end sequences were imported into the QIIME 2 (version 2023.7) pipeline [31] and were then merged and denoised with DADA2 [32]. The retrieved feature table and feature sequences of DADA2 were subsampled to the lowest sequence number (30,000) for each sample and subjected to downstream analysis, including diversity calculations and taxonomy annotations [33]. The taxonomic annotation of representative sequences was performed using a Naive Bayes classifier referenced against the SILVA release 138 database. Phylogenetic tree construction was achieved by aligning representative sequences with SILVA reference sequences using MAFFT, followed by maximum-likelihood tree inference via FastTree (v2.1.11) under the evolutionary model [34]. Alpha diversity indices and beta diversity metrics (weighted/unweighted UniFrac; Bray–Curtis measure) were subsequently calculated to characterize the microbial community structure. Microbial functional gene prediction was conducted using PICRUSt2, followed by the mapping of predicted genes to the KEGG database to generate KEGG Orthology (KO) abundance profiles [35,36].

### 2.3. Statistics and Visualization

Data analysis and visualization were carried out in R 4.2.3 (version 4.2.3) [37]. Differences in alpha diversity indices among the microbial communities were analyzed using Welch’s *t*-test with pairwise comparisons [38]. The analyses were performed in the R software environment (version 4.2.3) using the base stats package [39]. The beta diversity of microbial communities was analyzed using NMDS (non-metric multidimensional scaling) based on weighted UniFrac distances and was carried out with package vegan [39]. PERMANOVA (Permutational Multivariate Analysis of Variance) was employed to test for statistically significant differences in microbial community composition across distinct experimental scenarios [40,41]. *p*-values were calculated following 999 random permutations [42]. The results were visualized using R packages ggplot2 [43] and pheatmap [44].

## 3. Results

### 3.1. Microbial Community Composition During Incubation

A total of 65 bacterial phyla were identified across all samples. The ten most dominant phyla—Proteobacteria, Bacteroidota, Chloroflexi, Acidobacteriota, Planctomycetota, Desulfobacterota, Nitrospirota, Myxococcota, Verrucomicrobiota, and Cyanobacteria—together accounted for 95.3% to 95.5% of the total bacterial community. Among these, Proteobacteria showed the highest relative abundance, ranging from 33.7% to 38.5%.

**Figure 1:**
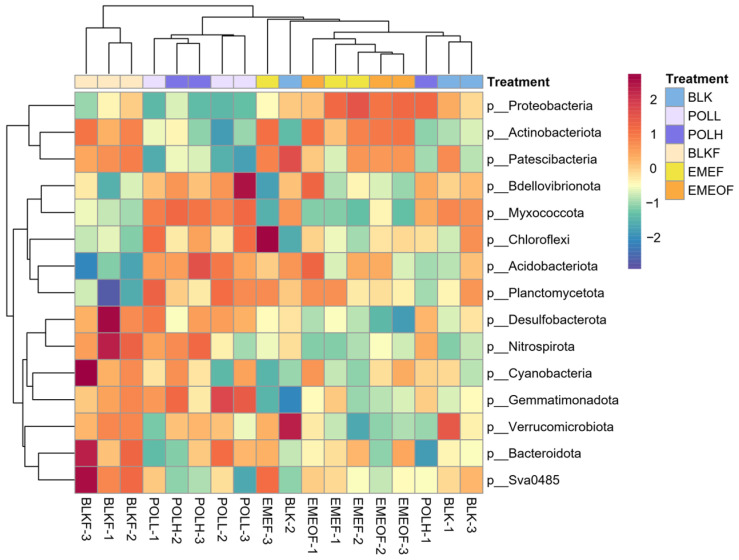
Phylum-level composition of the top 15 microbial taxa in sediment microbial communities across experimental groups on Day 14.

On Day 14, Proteobacteria were significantly enriched in thallium (Tl)-bearing floc groups (EMEF and EMEOF), exceeding the levels found in other treatment groups (Figure 1). By Day 3, the relative abundance of Myxococcota in the floc-containing groups (BLKF, EMEF, and EMEOF) declined to between 2.62% and 2.70% in EMEF and EMEOF, compared to the blank control group, which ranged from 2.91% to 3.22%. This difference in abundance increased over time. In the Tl shock-loading groups (POLL and POLH), the abundance of Nitrospirota increased from 3.15% to 3.43% in the control group to 3.21% to 3.78% in the POLL group and 3.60% to 4.05% in the POLH group, indicating a positive correlation with Tl contamination levels. Iron floc groups (BLKF, EMEF, and EMEOF) exhibited higher abundances of Actinobacteriota and Planctomycetota compared to other groups. Additionally, hierarchical clustering at the phylum level revealed distinct microbial structures in the Tl-free iron floc group (BLKF) when compared to the other treatments.

**Figure 2:**
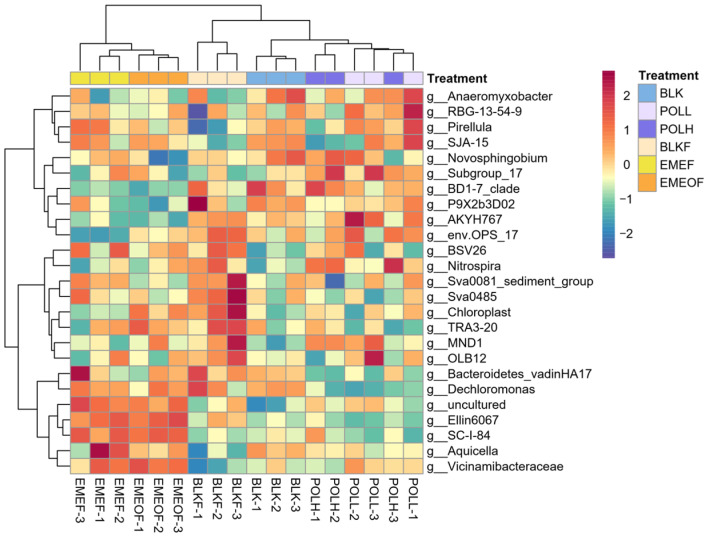
Genus-level composition of the top 25 taxa in sediment microbial communities across experimental groups on Day 14.

At the genus level, 1259 genera were annotated, with 145 showing average relative abundances exceeding 0.1%. Heatmap analysis of the top 25 genera (Figure 2) grouped the samples into two main clusters based on floc addition—low-concentration Tl shock loads and blank controls clustered closely together—indicating minimal divergence in their community composition. Genera *Ellin6067* and *SC_I_8* were found to be enriched in Tl-containing floc groups (EMEF and EMEOF), suggesting their potential resistance to heavy metals. In contrast, the abundance of Nitrospira in the shock-load groups (POLL and POLH) increased proportionally with the Tl concentration. At the same time, they remained unaffected in the iron floc treatments. In Tl-free floc groups, taxa including *BSV26*, *Bacteroidetes_vadinHA17*, *env.OPS 17* and *Novosphingobium* exhibited higher relative abundances. However, such patterns were not observed in Tl-bearing floc groups (EMEF and EMEOF), suggesting that these iron-associated taxa were also susceptible to Tl-induced stress despite their inherent iron metabolic dependencies.

### 3.2. Microbial Community Diversity

The alpha diversity indices, including faith_pd, the number of observed features, the Shannon index, and Pielou’s evenness, experienced slight fluctuations during incubation (Figure S1). The effect of Tl shock or flocs was insignificant, as indicated by Welch’s test (*p* > 0.05).

**Figure 3:**
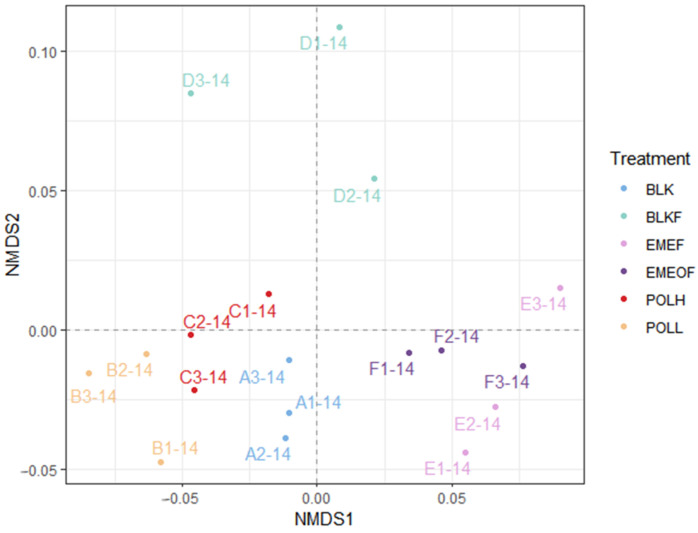
NMDS (non-metric multidimensional scaling) analysis of sediment microbial communities across experimental groups on day 14.

PERMANOVA analysis revealed statistically significant differences in microbial community structures among the emergency treatment groups on Day 14 (*p* < 0.05). Non-metric multidimensional scaling (NMDS) analysis, based on weighted UniFrac distance metrics, was performed on Day 14 on microbial communities to investigate the impacts of distinct emergency remediation measures on microbial community structures. The NMDS ordination of the Day 14 samples demonstrated distinct clustering patterns (Figure 3) as follows: (1) the Tl-free iron floc groups occupy the upper section of the plot, showing substantial separation from the other groups; (2) the blank controls and Tl shock-loading groups are clustered in the lower-left quadrant; and (3) the Tl-bearing floc groups are gathered in the lower-right quadrant. These results indicate that iron flocs significantly impacted microbial communities compared to Tl shock loads, with their influence potentially diminishing under conditions of Tl co-exposure.

### 3.3. Potential Function Shift in Microbial Community

**Figure 4:**
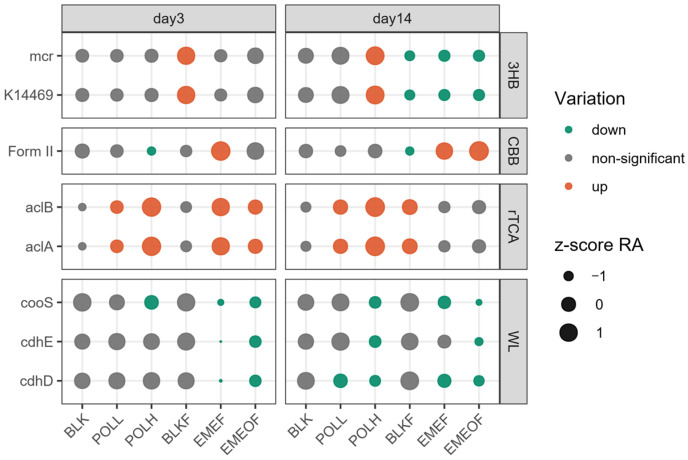
Carbon fixation pathway-associated genes on Days 3 and 14 (CBB, Calvin–Benson–Bassham cycle; rTCA, reductive citric acid cycle; WL, Wood–Ljungdahl pathway; 3HB, 3-hydroxypropionate bicycle).

The alpha diversity indices, including faith_pd, the number of observed features, and ShaFunctional gene prediction analyses, were conducted to investigate carbon-, nitrogen-, and sulfur-associated metabolic pathways. Four carbon fixation pathways were identified across the samples (Figure 4). Under Tl contamination, the abundance of genes linked to the Wood–Ljungdahl pathway declined. At the same time, the Reverse Tricarboxylic Acid Cycle (rTCA cycle) and Calvin–Benson–Bassham cycle (CBB cycle) were upregulated. In contrast, the Wood–Ljungdahl pathway remained unaffected in Tl-free iron floc groups. Temporal analysis revealed a progressive decline in the 3-hydroxypropionate cycle within the iron floc treatment groups (BLKF, EMEF, and EMEOF). Notably, no analogous suppression was observed under Tl shock-loading conditions, suggesting that the 3-hydroxypropionate cycle is predominantly modulated by iron dynamics rather than Tl toxicity.

**Figure 5:**
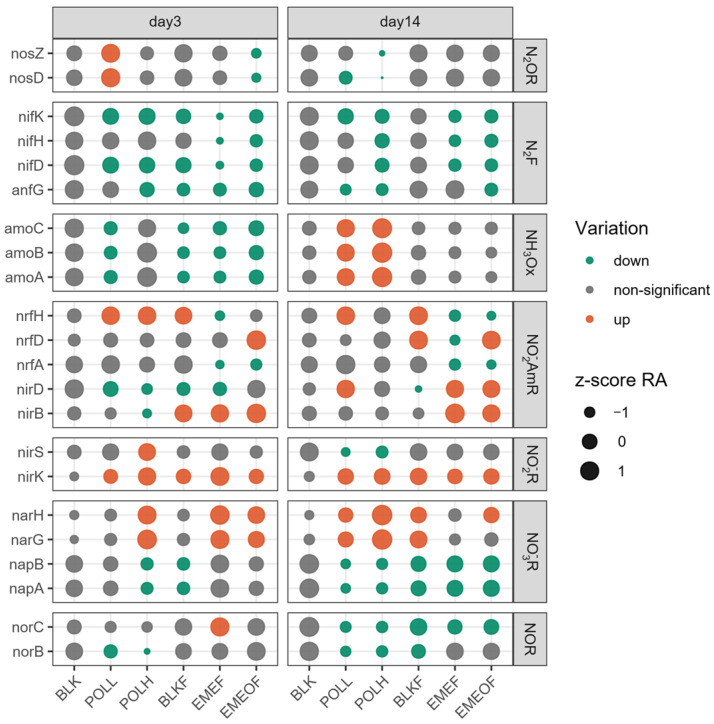
Nitrogen cycle-associated genes on Days 3 and 14 (N_2_OR, nitrous oxide reduction; N_2_F, N_2_ fixation; NO_2_^−^AmR, nitrite reduction to ammonia; NH_3_Ox, ammonia oxidation; NO_3_^−^R, nitrate reduction; NOR, nitric oxide reduction; NO_2_^−^R, nitrite reduction).

The nitrogen cycle-related processes identified in this study are illustrated in Figure 5. Under Tl contamination, genes associated with nitrogen fixation and nitric oxide reduction were suppressed in the Tl shock-loading and Tl-bearing floc groups. In contrast, genes linked to nitrite reduction and ammonia and dissimilatory nitrite reduction were enhanced. These findings suggest that Tl contamination exerts selective pressure on microorganisms harboring nitrogen cycle-related functional genes. Temporal dynamics analysis revealed more pronounced changes in nitrate reduction genes by Day 14: the relative abundance of Nap-associated genes decreased, while Nar-associated genes increased. Concurrently, in ammonia oxidation pathways, the relative abundance of Amo-associated genes in the Tl shock-loading groups transitioned from an initial decline to an upward trend. In contrast, the decline in Amo gene abundance in floc-treated groups ceased over time. Compared to Tl stress, ammonia oxidation processes may exhibit a more pronounced influence from iron (Fe) dynamics.

**Figure 6:**
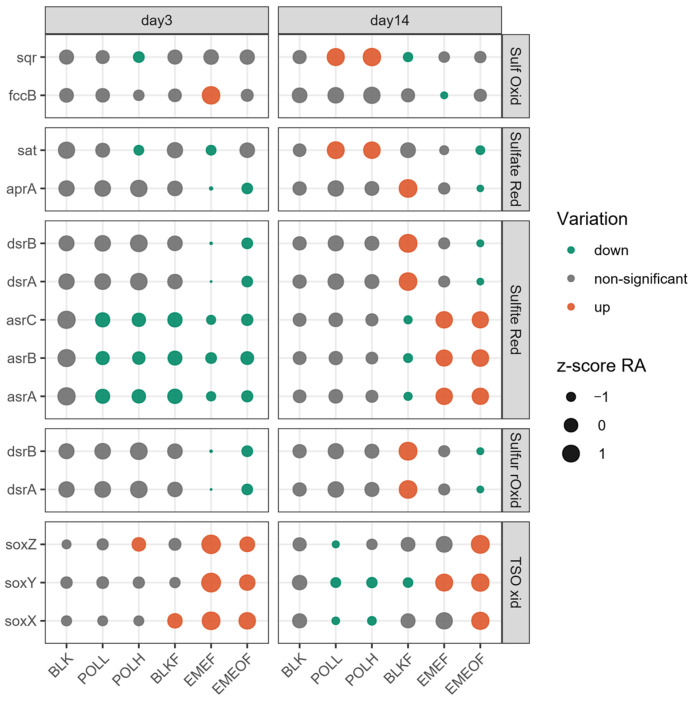
Nitrogen cycle-associated genes on Days 3 and 14 (Sulf Oxid, sulfide oxidation; Sulfite Red, sulfite reduction; TSO xid, thiosulfate oxidation; Sulfur rOxid, sulfur oxidation; Sulfate Red, sulfate reduction).

Under the combined effects of thallium (Tl) contamination and iron flocs, the relative abundance of the sulfite reduction pathway within the sulfur cycle showed an overall decline by Day 3 (Figure 6). Temporal analysis revealed the differing impacts of Tl and iron flocs on microbial communities by Day 14. In the Tl shock-loading groups (POLL and POLH), no significant changes were observed in the abundance of genes associated with sulfite reduction. In contrast, a continual decline was noted in the Tl-free floc groups. Conversely, the Tl-bearing floc groups (EMEF and EMEOF) experienced a shift from decreased to increased relative abundances of sulfite reduction-related genes. Additionally, the abundances of genes associated with thiosulfate oxidation decreased over extended periods in the Tl shock-loading groups (POLL and POLH); however, these genes exhibited an opposing trend in the Tl-bearing floc groups (EMEF and EMEOF). These results illustrate distinct patterns of modulation in the sulfur cycle depending on the emergency treatment strategies employed.

## 4. Discussion

### 4.1. Impacts of Thallium Shock Loading on Microbial Communities

The rapid expansion of mining, smelting, and industrial activities has led to frequent acute Tl contamination incidents, significantly impacting ecological systems in affected regions. Numerous studies have documented shifts in sediment microbial communities following sudden Tl pollution events in watersheds [26,45]. However, inherent limitations—such as temporal lag in field sampling and uncontrolled environmental variables—constrain the interpretation of these findings. This study simulated contamination scenarios under controlled laboratory conditions to investigate microbial community responses and feedback mechanisms under Tl shock loading and emergency remediation.

In Tl shock-loading groups, a positive correlation was observed between Tl exposure intensity and the relative abundance of Nitrospira. As a keystone nitrifying microorganism, Nitrospira mediates the oxidation of nitrite to nitrate and demonstrates remarkable adaptability across diverse environments, including heavy-metal-contaminated soil and aquatic systems [46,47]. Prior research indicates that Nitrospira mitigates metal-induced oxygen limitation by producing high-affinity hemoproteins, conferring robust tolerance to heavy metal stress [48], which aligns with our findings and underscores its resilience to Tl contamination.

Alterations in the microbial community structure are often accompanied by functional adaptations or impairments, particularly under heavy metal stress, where microbes modulate specific metabolic pathways to cope with environmental challenges [49]. Nitrate reduction, a critical nitrogen metabolism process, relies on key enzymes encoded by genes such as napA and napB (periplasmic nitrate reductase, NAP) and narG and narH (membrane-bound nitrate reductase, NAR) [50,51,52]. Previous studies have focused on three types of microorganisms, each with unique nitrate reductases [53]. These studies showed that the growth patterns of these microorganisms varied significantly under cadmium contamination [53]. This suggests a possible link between nitrate reduction genes and the ability of microorganisms to adapt to heavy metal stress [53,54]. In this study, Tl-contaminated groups preferred membrane-bound nitrate reductases, potentially linked to Tl detoxification and tolerance mechanisms. Notably, during the initial phase of Tl exposure, microbial communities suppressed the upregulation of genes associated with nitrogen fixation (NF) and denitrification (NR). Nitrogen fixation is an energetically demanding process, with each reduction in nitrogen gas (N₂) requiring the consumption of 16 ATP molecules [55]. According to previous research reports, when heavy metals contaminate the soil, limited energy resources are prioritized for maintaining basic cellular functions rather than energy-intensive proliferation-related metabolic pathways such as nitrogen fixation [56]. This energy redistribution effect and the direct inhibition of heavy metals on nitrogenase (such as the binding of metal ions to the active center of the enzyme) form dual pressure, eventually leading to a significant decrease in the abundance of nifD/nifH/nifK genes in the soil of the contaminated area [56]. Under heavy metal stress, the microbial nitrogen cycle community was adapted from an energy-intensive metabolic model to a low-energy consumption survival strategy [57,58]. The optimization of microbial nitrogen cycle strategies can improve metal detoxification mechanisms, thereby promoting environmental sustainability and ecological balance.

Compared to field observations at comparable Tl concentrations (20–100 μg/kg), laboratory-simulated communities exhibited attenuated structural responses to Tl [21,54]. This discrepancy likely arose from the inherent complexity of natural ecosystems—including unmodeled variables such as synergistic stressors and dynamic aquatic biogeochemical cycling—that cannot be fully replicated in controlled experimental systems.

### 4.2. Effects of Thallium-Bearing Flocs on Microbial Communities

NMDS analysis revealed that Tl-free iron floc groups occupied distinct positions from other treatment clusters, indicating that emergency treatment-generated iron flocs substantially impacted the microbial community structure. Iron (Fe), an essential trace element for bacterial growth and metabolism, has been shown to enhance microbial proliferation and metabolic cycling [59]. It has been reported that most species within the phylum Actinomycetes can acquire energy by transferring electrons from the cell membrane to reduce Fe(III) via various mechanisms [60]. In this study, adding iron(III) flocs enhanced the relative abundance of the phylum Actinomycetes, suggesting a potential association between Actinomycetes and the microbial utilization of Fe(III) reduction as an energy source. Moreover, numerous existing studies have demonstrated that Actinomycota not only mediates the redox processes of heavy metals but also exhibits diverse functionalities and strong adaptability (e.g., leaching rare earth elements and degrading recalcitrant organic compounds), thereby offering broad prospects for environmental pollution control and remediation [61,62]. Based on previous research, the metabolic products produced by iron-associated microorganisms help adsorb heavy metals in soil through electrostatic interactions and chelation [23,63]. This process reduces the bioavailability and toxicity of these heavy metals. This finding aligns with the results of the current study, which showed that the Actinomycetes phylum was notably prominent in the group containing Tl flocs.

Iron amendments also significantly modulated biogeochemical cycling. Existing studies report that iron suppresses nap-associated gene abundance and inhibits denitrification processes [64], which is consistent with our results. The addition of iron flocs led to a decrease in the relative abundance of the amoA/B/C gene. This finding indicates that nitrification in the sediment was suppressed, which is consistent with previous research on soil studies [65]. Furthermore, over time, the inhibitory effect weakened gradually, likely due to the adaptive evolution of the microbial community in the sediment. In this study, Fe(III) floc addition downregulated the 3-hydroxypropionate bicycle pathway in carbon fixation and sulfite reduction in sulfur cycling. While the mechanisms underlying iron floc-driven biogeochemical alterations post-remediation remain poorly characterized, emerging evidence highlights microbially mediated iron cycling coupled with diverse elemental cycles [66,67]. For instance, Fe-ammonia oxidation (Feammox) under Fe-N coupling may contribute to nitrogen loss in aquatic systems, while Fe-C interactions can suppress methanogenesis and mitigate greenhouse gas emissions [67,68]. These findings underscore the non-negligible ecological impacts of secondary floc formation on microbial communities and the associated environmental risks following emergency interventions.

### 4.3. Influence of Thallium-Free Flocs on Microbial Communities

Iron (Fe), as a redox-active element in soil and sediments, critically regulates the speciation, mobility, and bioavailability of heavy metals (e.g., Cr, As, Cd, Tl) through diverse physicochemical mechanisms, thereby modulating their ecological toxicity [23,59,69,70]. Studies demonstrate that iron oxides provide abundant reactive sites for heavy metal adsorption via surface hydroxyl groups and coordination bonding, effectively altering their mobility and bioavailability [71,72,73]. However, the structural destabilization of iron flocs or competition between Fe^3+^ and heavy metals for organic ligand-binding sites may trigger the re-release of immobilized contaminants, posing secondary environmental risks [74,75].

Proteobacteria, renowned for their environmental adaptability, have been identified as dominant taxa in multiple heavy metal-contaminated soils and sediments [26,45,76], which is consistent with observations in Tl-bearing floc groups. This phylum exhibits resistance to heavy metal stress and demonstrates the potential for bioremediation through mechanisms such as adsorption and redox transformations [77,78,79]. Furthermore, at lower taxonomic levels, the bacteria *Ellin6067* and *SC_I_84*, which exhibit tolerance to Tl contamination, are classified under the phylum Proteobacteria. Notably, the heavy metal tolerance of *Ellin6067* has been documented in numerous studies [80,81].

Investigating functional gene expressions in microbially mediated nitrogen and sulfur cycles is pivotal for elucidating biogeochemical perturbations under heavy metal contamination [49,79]. Sulfate-reducing and oxidizing microorganisms, for instance, mitigate metal mobility and toxicity via sulfide precipitation and ligand complexation [82,83]. This study’s upregulation of sulfate reduction- and oxidation-associated genes in Tl-contaminated experimental groups aligns with these findings. Notably, under the combined stress of iron flocs and Tl, the inhibition of nitrate reductase genes (napABC) in Tl-bearing floc groups was less pronounced than in Tl shock-loading groups. This iron floc-mediated enhancement of napABC gene activity corroborates prior reports in iron-rich sludge systems [84]. Current evidence suggests that Tl-bearing flocs exert multifactorial dependencies on nitrogen cycling, with mechanisms intricately linked to Tl concentration gradients, dissolved oxygen levels, and organic carbon availability. The precise pathways (e.g., Feammox and denitrification coupling) and molecular drivers underlying these interactions require further exploration through integrated multi-omics and in situ microcosm approaches.

According to the MNDS analysis of the microbial community on the 14th day, the difference in microbial composition between the Tl shock load group and the blank control group was minimal. This suggests that diluting the water as an emergency response during sudden events has a relatively limited impact on the microbial community. In contrast, the Tl-free iron flocs group showed significant differences, indicating that iron flocs significantly alter the structure of the microbial community. When assessing ecological risks related to sudden pollution incidents in basins, the effects of iron flocs should be carefully evaluated and prioritized. It is crucial to thoroughly assess actual pollution situations and to scientifically design emergency response measures and control strategies for adding coagulant flocs. This approach will help achieve both effective pollution mitigation and competent ecological risk management.

## 5. Conclusions

This study investigates the impacts of different emergency treatment measures on microbial communities’ structural and functional dynamics following acute Tl contamination, providing a comprehensive assessment of the potential risks associated with remediation strategies. Based on 16S rRNA gene sequencing, we identified treatment-specific shifts in microbial community composition. The emendation of Fe(III) floc led to an increase in the relative abundance of Actinobacteriota. Meanwhile, Nitrospira and Proteobacteria exhibited distinct tolerances to Tl shock and Tl floc stress, respectively. Tl pollution inhibited a reduction in nitric oxide and nitrogen fixation while increasing the relative abundance of napA/B genes and decreasing the relative abundance of narG/H genes involved in nitrate reduction. In Tl shock treatments, genes associated with sulfate reduction were downregulated, while in Tl-floc groups, these genes were upregulated after contamination. These differences mainly stemmed from variations in Tl exposure levels and the synergistic interactions between Tl and iron under different remediation scenarios.

## Data Availability

Raw sequence data were deposited in NCBI Sequence Read Archive (SRA) under the project number PRJNA1230566 at Available online: https://www.ncbi.nlm.nih.gov/sra/PRJNA1230566 (accessed on 11 April 2025).

## Supplementary Materials

The following supporting information can be downloaded at https://www.mdpi.com/article/10.3390/microorganisms13061336/s1. Text S1: Emergency coagulation and precipitation treatment; Table S1: Physicochemical properties of the Yexi River; Figure S1: Differences in microbial diversity.
