# Supplementary material for: Impacts of Emergency Treatments on Sediment Microbial Communities Following Sudden Thallium Contamination Events: A Microcosm Study
Source: Microorganisms. 2025 Jun 9;13(6):1336. doi: 10.3390/microorganisms13061336 (PMC12195376; doi:10.3390/microorganisms13061336)
Supplement: Supplementary file 1 [file microorganisms-13-01336-s001.zip › microorganisms-3612378-supplementary.pdf]

Text S1. Emergency coagulation and precipitation treatment

Coagulation and Sedimentation

1. Adjust the pH value of the raw water to 8.0–9.0 using sodium hydroxide.
2. Pour 600 mL of raw water into a 1 L beaker.
3. Add 3 mL of a 10 g/L ferric sulfate polymer solution and stir at 200 r/min for 3 minutes.
4. Reduce the stirring speed to 40 r/min and continue stirring for 15 minutes.
5. Allow the mixture to settle for 30 minutes to obtain iron flocs without thallium.
6. Replace the 600 mL of raw water with 600 mL of laboratory-simulated thallium-contaminated wastewater with a thallium concentration of 100 μg/L, and repeat the above coagulation and sedimentation experiment to obtain thallium-containing iron flocs.

Emergency Pre-oxidation and Coagulation

1. Adjust the pH value of the raw water to 8.0–9.0 using sodium hydroxide.
2. Pour 600 mL of laboratory-simulated thallium-contaminated wastewater with a thallium concentration of 100 μg/L into a 1 L beaker.
3. Add 1 mL of a 1 g/L potassium permanganate oxidant and allow the mixture to pre-oxidize for 30 minutes.
4. Add 3 mL of a 10 g/L ferric sulfate polymer solution and stir at 200 r/min for 3 minutes.
5. Reduce the stirring speed to 40 r/min and continue stirring for 15 minutes.
6. Allow the mixture to settle for 30 minutes to obtain iron flocs without thallium.

Table S1. physicochemical properties of the Yexi River

Do (mg/L)	μ (μs/cm)	pH	TN (ug/mL)	$NO_3{}^-$ (ug/mL)	$NH_4{}^+$ (ug/mL)	TP (ug/mL)	CODMn (mg/L)
8.8	337	6.53	2.08	0.69	0.553	0.098	3.8

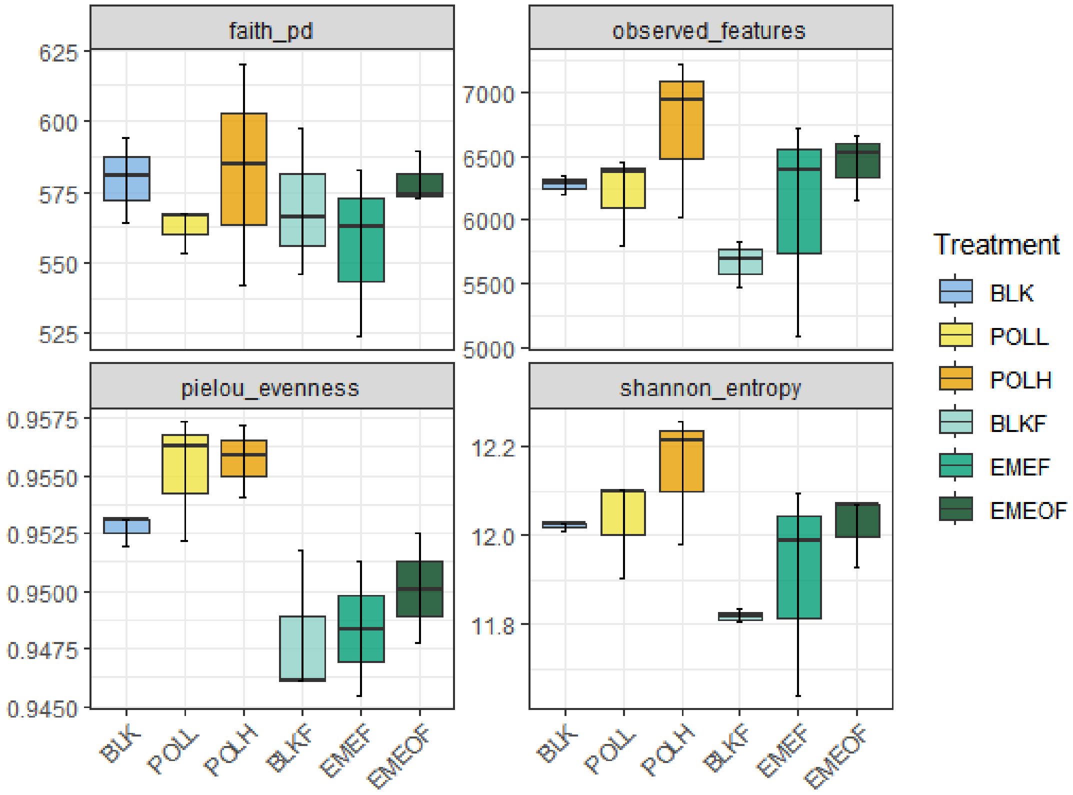

Figure S1. Differences in microbial diversity
